# A multidisciplinary, phased nursing strategy for skin and mucosal management in a pediatric case of toxic epidermal necrolysis with respiratory failure: a case report

**DOI:** 10.3389/fped.2026.1759262

**Published:** 2026-01-20

**Authors:** Cheng Yang, Yang Wen, Peijin He, Yanling Dong, Yunjie Feng

**Affiliations:** 1 Department of Pediatric Intensive Care Unit Nursing, WCSUH-Tianfu·Sichuan Provincial Children’s Hospital, Meishan, China; 2 Department of Pediatric Intensive Care Unit Nursing, West China Second University Hospital, Sichuan University, Meishan, China; 3 Key Laboratory of Birth Defects and Related Diseases of Women and Children, Sichuan University, Ministry of Education, Chengdu, China

**Keywords:** case report, multidisciplinary team, pediatrics, respiratory failure, skin care, toxic epidermal necrolysis

## Abstract

**Introduction:**

Toxic Epidermal Necrolysis (TEN) is a life-threatening dermatologic emergency, with particularly high morbidity and mortality in children due to their vulnerable skin barrier and propensity for systemic complications. When TEN is further complicated by respiratory failure, the competing demands of airway management and skin preservation pose a significant therapeutic challenge.

**Case report:**

We report a case of a 6-year-old boy with TEN involving 70% body surface area and concurrent respiratory failure. The patient required endotracheal intubation and mechanical ventilation, which exacerbated facial skin injury. A structured, multidisciplinary, and phased nursing protocol was implemented, integrating respiratory support with meticulous skin, ocular, oral, and urogenital care.

**Interventions & outcomes:**

A coordinated team comprising pediatric intensive care, dermatology, infectious diseases, and nutrition specialists guided management. Skin care was staged according to wound healing phases, utilizing non-adhesive dressings, topical recombinant bovine basic fibroblast growth factor (bFGF) gel, and innovative tube-securement techniques. Systemic and mucosal care protocols were rigorously applied. The patient achieved complete re-epithelialization by day 35, was successfully extubated, and discharged in stable condition on day 46 with no major sequelae.

**Conclusion:**

This case demonstrates that a structured, phase-based, and multidisciplinary nursing approach can effectively balance life-sustaining interventions with tissue preservation in severe pediatric TEN. The strategy highlights the importance of adaptive wound staging, trauma-minimizing techniques, and proactive mucosal protection, offering a replicable framework for similar critical care scenarios.

## 1 Introduction

Toxic Epidermal Necrolysis (TEN) is a life-threatening severe cutaneous and mucosal adverse reaction characterized by extensive epidermal detachment (1). TEN is particularly dangerous in children; due to their immature skin barrier and higher body surface area-to-weight ratio, they are more prone to fluid loss, electrolyte imbalances, and secondary infections, with a mortality rate reaching 30%–50% (2).

When TEN involves the respiratory mucosa and leads to respiratory failure, the condition becomes even more complex and critical, posing extreme challenges for both life-sustaining interventions and wound care (3). Essential procedures for airway management, such as endotracheal intubation, can further damage the already compromised skin around the mouth and face, creating a therapeutic dilemma. Therefore, coordinating respiratory support with meticulous cutaneous care in such critically ill children remains a key challenge in pediatric intensive care (4).

This article reports the case of a 6-year-old boy with TEN involving up to 70% of his BSA that was complicated by respiratory failure. Through multidisciplinary collaboration, we implemented a comprehensive, phased cutaneous and mucosal management plan, which successfully addressed this therapeutic conflict (5). This report aims to detail the nursing management of this case to provide a reference for managing other critically ill children with similar conditions (6).

## 2 Case report

A 6-year-old boy was admitted to the Pediatric Intensive Care Unit (PICU) on July 23, 2025, with a two-day history of fever, which had acutely worsened over the preceding 24 h due to the emergence of a generalized rash and blistering. On admission, physical examination revealed hyperpyrexia (40.2 °C), tachycardia (139 bpm), tachypnea (28 breaths/min), and normotension (118/65 mmHg). The child was conscious. Cutaneous examination showed widespread papules and flaccid blisters over the face, neck, trunk, and extremities, with confluent areas predominantly on the chest, back, and face, involving approximately 70% of his body surface area (BSA). Additional findings included bilateral conjunctival injection with purulent discharge, perioral fissuring, and exudative erosions around the urethral meatus and perianal region. Peripheral perfusion was preserved.

Based on the febrile presentation, leukopenia (1.15 × 10^9^/L), elevated inflammatory markers (procalcitonin 1.361 ng/mL, CRP 35 mg/L), and imaging evidence of pulmonary infiltrates, the admission diagnoses were Toxic Epidermal Necrolysis (TEN), severe pneumonia, and suspected sepsis. The systemic inflammatory response was considered attributable either to the cytokine storm associated with TEN or to a concurrent bacterial infection. The parents reported that the child had received an oral herbal preparation and an herbal bath two days prior to fever onset, suggesting a probable drug-induced etiology. The SCORTEN score on admission was 3 (based on tachycardia, BSA involvement >70%, and a serum bicarbonate level of 17.0 mmol/L), predicting an approximate mortality risk of 35%. Other relevant laboratory abnormalities included thrombocytopenia (116 × 10⁹/L), hyponatremia (128.2 mmol/L), and hypokalemia (3.6 mmol/L).

## 3 Initial management and clinical deterioration

**Figure 1:**
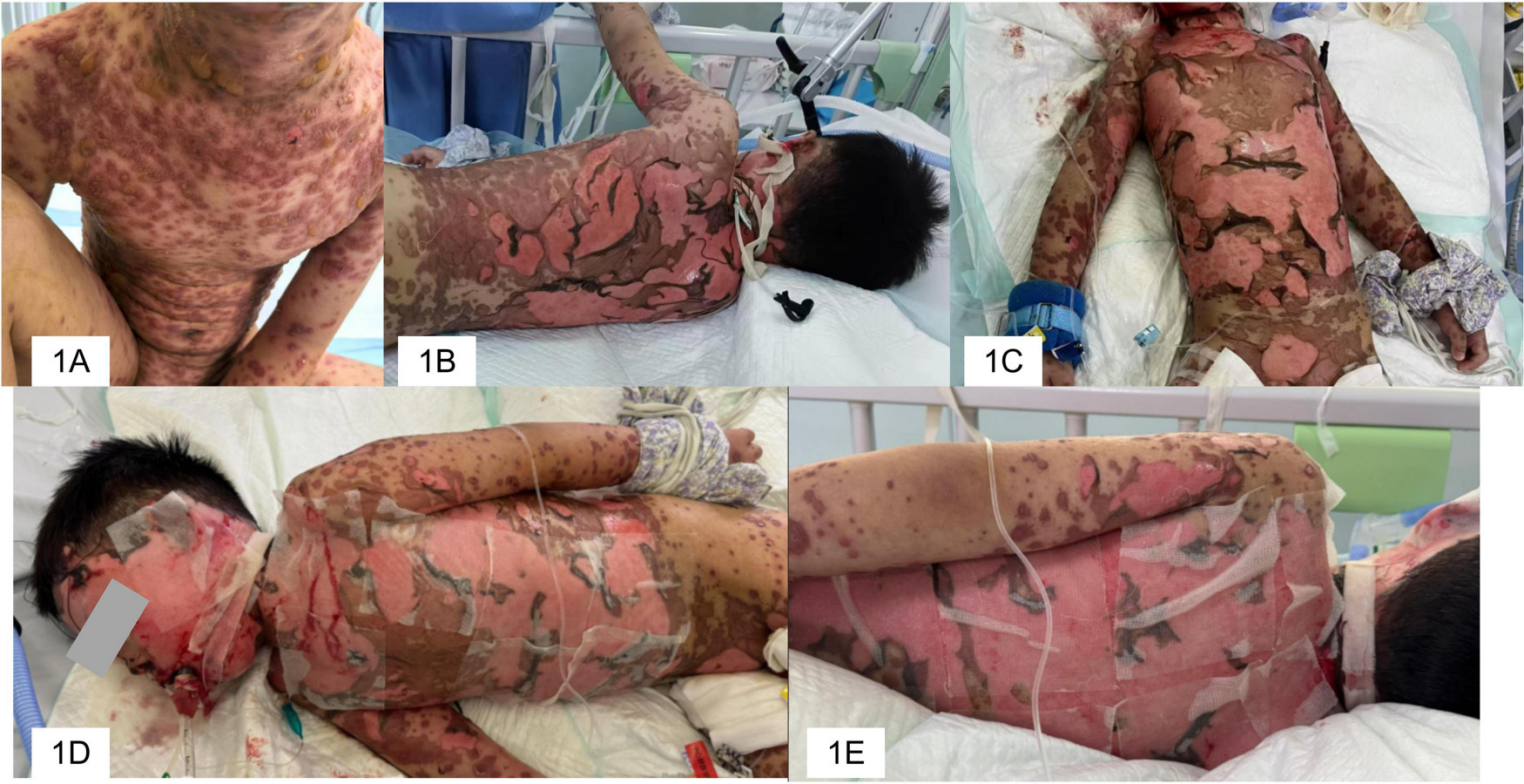
Panel **(A)** the patient presented with a severe generalized rash on the day of admission. Panels **(B,C)** Extensive skin desquamation observed on day 3 of hospitalization. Panels **(D,E)** Details of the daily skin care procedures.

Initial management included high-flow nasal cannula oxygen therapy, placement of central venous and blood purification catheters, and immediate initiation of empirical anti-infective therapy (ceftazidime, vancomycin, azithromycin, and acyclovir), anti-inflammatory agents, and continuous renal replacement therapy. However, within five hours of admission, the blistering progressed rapidly, resulting in widespread epidermal detachment clinically estimated to involve >90% BSA. This was accompanied by respiratory distress necessitating endotracheal intubation and mechanical ventilation. The intubation procedure inflicted further trauma to the compromised facial skin, causing extensive epidermal loss that was immediately covered with petrolatum gauze (Figure 1).

## 4 Hospital course and complications

By hospital day 3, the epidermis over the chest, abdomen, back, and head and neck had fully detached, accompanied by considerable hemorrhagic exudate. During this critical phase, the patient developed ventilator-associated pneumonia. Sputum culture (obtained around hospital day 9) and subsequent bronchoalveolar lavage (BAL) fluid metagenomic sequencing confirmed infection with carbapenem-resistant Acinetobacter baumannii (CRAB) and Enterobacter cloacae complex, definitively establishing the diagnosis of bacterial sepsis complicating TEN. Management was escalated to include local hemostasis, antimicrobial therapy tailored to the multidrug-resistant pathogens, continued wound coverage with petrolatum gauze, and topical application of epidermal growth factor.

## 5 Bronchoscopic evaluation

On hospital day 17 (August 9, 2025), bedside bronchoscopy was performed due to persistent respiratory distress and suspected airway compromise. Direct laryngoscopy revealed edematous and erythematous pharyngeal and laryngeal mucosa without evident pseudomembrane formation. Bronchoscopic examination showed a smooth and sharply defined carina, clearly visible tracheal cartilage rings, and diffusely congested but intact tracheobronchial mucosa. The left bronchial tree contained copious white, tenacious secretions and scattered mucosal bleeding points in the basal segments, while the right bronchial tree had minimal thin secretions. No airway stenosis or granulation tissue was observed. BAL fluid later confirmed infection with CRAB and Enterobacter cloacae complex.

## 5 Systemic pharmacotherapy

Guided by the multidisciplinary team, systemic pharmacotherapy comprised intravenous immunoglobulin (IVIG) at a total dose of 2 g/kg administered over two days, intravenous methylprednisolone initiated at 2 mg/kg/day with subsequent tapering, and three sessions of therapeutic plasmapheresis. Notably, neither TNF-α inhibitors nor cyclosporine were administered, primarily due to concerns regarding infection risk and hemodynamic instability in the acute phase.

## 6 Recovery and discharge

By hospital day 10, active bleeding had subsided and granulation tissue began to form. New epidermal coverage was observed over most body surfaces by day 15. The endotracheal tube was successfully removed on day 20, after which nursing care focused intensively on facial skin rehabilitation. By day 30, the healed skin had formed a black eschar, which was gently debrided. Complete re-epithelialization was achieved by day 35, with residual post-inflammatory hyperpigmentation. Finally, on day 46, with all vital signs stable and hyperpigmentation showing signs of fading, the patient was discharged in stable condition for outpatient follow-up.

## 7 Nursing interventions

The patient was managed in a Pediatric Intensive Care Unit (PICU) in accordance with an institutional burn care protocol. Nursing staff were trained in burn wound management, and all skin, ocular, and mucosal interventions were guided by established burn care principles and published TEN-specific guidelines (7–9).

### 7.1 Multidisciplinary collaborative management

A structured multidisciplinary team (MDT) was assembled to oversee the patient' s care, comprising specialists from the PICU, Dermatology, Infectious Diseases, and Nutrition departments. The team operated under a coordinated model of “specialist collaboration with clear role allocation.” Specifically, the PICU team was responsible for hemodynamic stabilization and organ support; the Dermatology team conducted daily wound assessments and performed targeted interventions to promote tissue regeneration; the Infectious Diseases department led infection prevention and control, including antimicrobial stewardship; and the Nutrition team provided individualized caloric and high-protein support to meet metabolic demands. Additionally, a senior PICU nurse with over five years of experience was assigned as the primary care nurse. This nurse was responsible for performing specialized procedures, continuously monitoring vital signs and skin recovery, providing psychological support to the child and family, and ensuring overall treatment safety and quality of life throughout hospitalization. The phase-based, multidisciplinary nursing management protocol is summarized in Table 1.

**Table 1:** Phase-Based, multidisciplinary nursing management protocol.

Phase and Timing	Primary Goals	Key Interventions	Multidisciplinary Team (MDT)
Phase 1: Acute Admission (Days 1–2)	Stabilization and protection.	Skin: Saline, petrolatum gauze, air mattress.	PICU Nursing (lead), Dermatology/Urology (consult).
		Eyes: Initial saline irrigation and lubrication.	
		Airway: HFNC monitoring.	
		Urogenital: Silicone Foley catheter insertion.	
Phase 2: Intubation and Critical Care (Days 3–20)	Balance support and healing; infection control.	Cleansing: Sequential saline irrigation followed by diluted povidone-iodine wet compresses (every 6–8 h).	PICU Nursing (lead), Dermatology (daily assessment), Ophthalmology/ID/Pharmacy (consult).
		Skin: q6–8 h saline/povidone-iodine cleansing; non-adhesive tube Securement (sterile rope); topical bFGF gel + infrared.	
		Eyes: q6 h irrigation and separation; anti-adhesion gel; q2 h antibiotic-steroid drops.	
		Airway/Oral: Structured BID oral care (two-nurse, cuff pressure control, chlorhexidine); bronchoscopy (Day 17).	
		Urogenital: q4 h catheter rotation; perineal care.	
Phase 3: Post-Extubation and Rehabilitation (Days 21–46)	Complete healing and functional recovery.	Skin: Continued bFGF until Day 35; facial alternating dressings (bFGF/silver-ion); eschar management with saline soaks.	PICU Nursing, Dermatology/Ophthalmology (follow-up), Nutrition/Rehab (consult).
		Eyes/Oral/Airway: Maintenance care & functional monitoring.	
		Urogenital: Catheter removed (Day 22); voiding function monitored.	
Systemic and Nutritional Support (Across all phases)	Infection control, metabolic and tissue repair support.	Tailored antimicrobials (e.g., for CRAB), IVIG (2 g/kg), corticosteroids, plasmapheresis, high-protein nutrition.	Infectious Diseases, Pharmacy, Nutrition Team, PICU Medicine.

### 7.2 Phased skin care: A structured and individualized protocol

The management of extensive cutaneous involvement constituted the central nursing challenge. While established guidelines emphasize avoiding further injury through local protection, non-adherent dressings, maintenance of a moist wound environment, and minimization of dressing changes, our team confronted an additional pharmacological concern (7, 10–12). Given the drug-induced etiology and the exceptionally large body surface area affected, the use of topical agents with systemic absorption potential—such as mupirocin ointment or silver-containing dressings—posed a risk of cumulative toxicity (13, 14). Consequently, after multidisciplinary evaluation, recombinant bovine basic fibroblast growth factor (bFGF) gel was selected as the primary topical wound-healing agent owing to its favorable local safety profile. The complexity of care was further heightened by facial skin trauma exacerbated by endotracheal intubation. Accordingly, a detailed, phase-based skin care protocol was implemented, structured into two main periods.

#### 7.2.1 Care during endotracheal intubation

##### 7.2.1.1 Whole-body protection

In addition to standard pressure-injury prevention, the patient was placed on an air-fluidized mattress and repositioned every 2 h, avoiding the supine position to reduce shear and friction. All open wounds were covered with petrolatum gauze. A customized protective frame was constructed using sterile ropes and bed rails to suspend the body above the linens, thereby preventing direct contact and providing both insulation and protection against adhesion.

##### 7.2.1.2 Facial tube securement and protection

**Figure 2:**
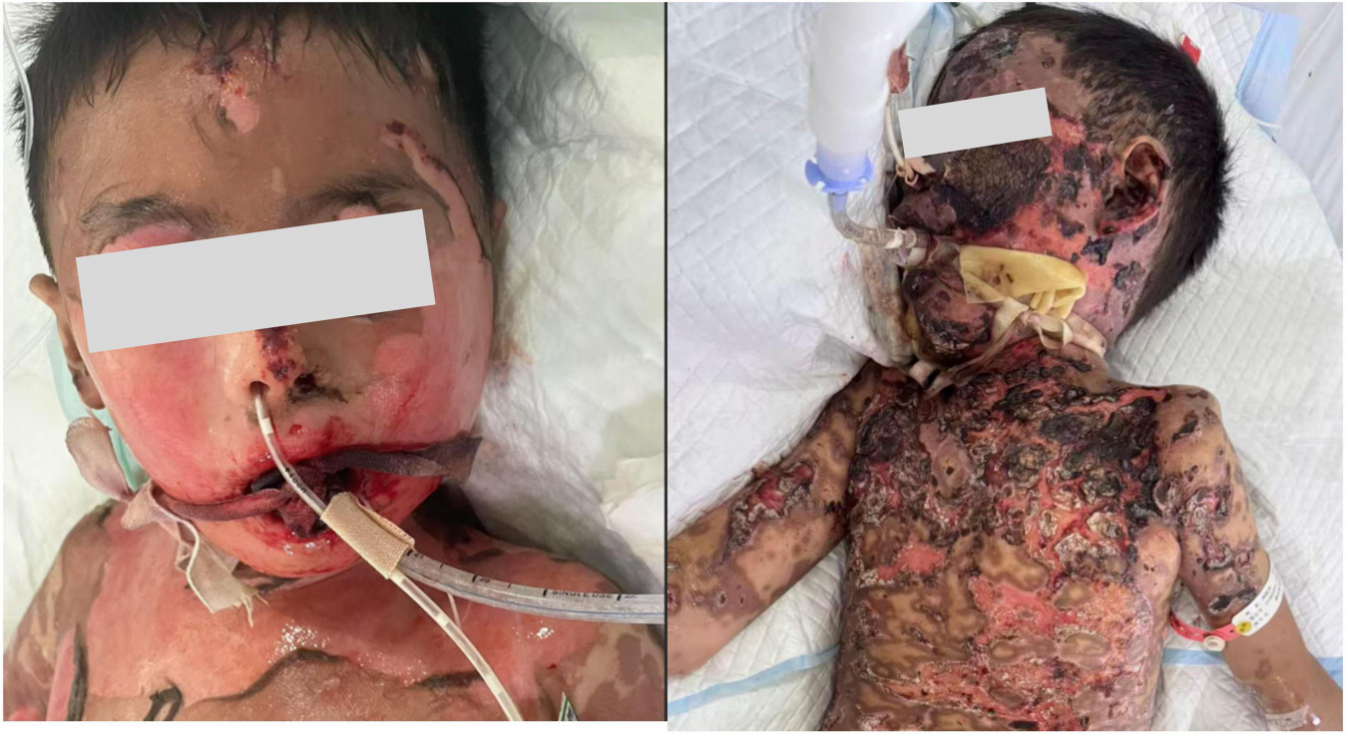
Panel **(A)** condition of the skin on the bilateral cheeks and the individualized securement of the endotracheal tube. Panel **(B)** Significant bleeding during the intubation period, leading to the formation of blood crusts.

Because adhesive tape was contraindicated due to extensive facial epidermal loss, the endotracheal tube was secured using a sterile rope looped twice around the tube and tied behind the neck. Local facial wounds were disinfected and covered with petrolatum gauze, with topical prothrombin powder applied daily to control oozing (Figure 2A).

##### 7.2.1.3 Cleansing and disinfection

Before the appearance of granulation tissue, wounds were irrigated with normal saline and treated with diluted povidone-iodine wet compresses for 20 min every 6 h. After granulation tissue began to form, irrigation was performed every 8 h using a sequential protocol: normal saline, followed by sterile water, and finally diluted povidone-iodine (9:1 ratio).

##### 7.2.1.4 Healing promotion

Recombinant bovine basic fibroblast growth factor (bFGF) gel was applied as the primary topical agent at a concentration of 210 IU/cm^2^. Initially, the gel was spread evenly onto petrolatum gauze, which was then placed over the wounds and changed every 6 h. Once granulation tissue developed, the gel was applied directly to the wound bed every 8 h. This regimen was supplemented with continuous infrared lamp therapy to enhance local microcirculation.

#### 7.2.2 Post-Extubation skin care

##### 7.2.2.1 Whole-body skin

The protocol of cleansing, disinfection, and topical bFGF application was continued until complete re-epithelialization was achieved.

##### 7.2.2.2 Facial skin

Following hemostasis with prothrombin powder, facial wounds were covered alternately with bFGF gel and silver-ion dressings twice daily. Silicone foam dressings were used between applications to manage exudate.

##### 7.2.2.3 Eschar management

After the formation of black eschar, daily normal saline soaks were applied to gently soften and debride the crust, thereby facilitating epidermal exposure (Figure 2B).

#### 7.2.3 Outcome

This structured nursing protocol supported a predictable healing trajectory. Granulation tissue emerged by hospital day 10, followed by new epidermal coverage by day 15. After extubation on day 20, intensified facial care led to well-formed eschar by day 30, with complete eschar separation and re-epithelialization achieved by day 35. These results confirm the efficacy of this individualized, phase-based skin management strategy.

### 7.3 Eye care

Ocular involvement is a major source of morbidity in toxic epidermal necrolysis (TEN), with complication rates reported between 40% and 84% in pediatric cases (15). During the critical acute phase, sight-threatening manifestations—including conjunctival injection, pseudomembrane formation, epithelial defects, and eyelid adhesions—may be overshadowed by life-threatening systemic instability. Without timely intervention, these can progress to corneal ulceration, perforation, and permanent vision loss. Evidence emphasizes that early, proactive management combining saline irrigation with targeted pharmacotherapy is essential to mitigate long-term sequelae.

Upon admission, our patient exhibited severe periocular involvement: bilateral eyelid skin detachment, significant purulent discharge, pseudomembrane formation, and swollen, adherent eyelids that prevented voluntary eye opening. Following multidisciplinary consultation, a structured ophthalmological care protocol was implemented:

#### 7.3.1 Debridement and cleansing

Every 6 h, the eyelids were gently separated using sterile cotton-tipped applicators, followed by irrigation with normal saline to remove secretions and necrotic debris.

#### 7.3.2 Moisturization and anti-adhesion

To prevent symblepharon, after each irrigation a sterile glass rod or cotton-tipped applicator was used to gently separate the palpebral and bulbar conjunctiva. Deproteinized calf blood extract eye gel and sodium hyaluronate drops were applied alternately, ensuring even distribution over the corneal surface and fornices.

#### 7.3.3 Anti-Inflammatory and anti-infective therapy

Tobramycin/dexamethasone eye drops were administered every 2 h to control local infection and inflammation.

#### 7.3.4 Periocular wound care

Every 6 h, the periocular skin was treated with a 20-minute wet compress using a solution of sterile water and povidone-iodine (9:1 ratio). Subsequently, recombinant bovine basic fibroblast growth factor (bFGF) gel was thinly applied onto petrolatum gauze and placed over the denuded periorbital area to promote epithelialization.

Through this systematic regimen, the patient regained voluntary eye opening by hospital day 7. Visual function was fully restored by day 12, demonstrating the effectiveness of this coordinated, multi-targeted nursing approach in preserving ocular integrity and function.

### 7.4 Oral intervention

Oral mucosal involvement is a hallmark of toxic epidermal necrolysis (TEN), often presenting as painful erosions or necrosis that may extend to the pharynx and other mucosal surfaces, thereby increasing the risk of secondary infection and systemic inflammation (16, 17). In this critically ill pediatric patient, oral endotracheal intubation was required for mechanical ventilation, while active bleeding and extensive denudation were observed in the oral and perioral mucosa. The presence of oral malodor further indicated a high risk of local infection. A systematic protocol was therefore implemented, integrating mucosal assessment with structured preventive care.

#### 7.4.1 Baseline mucosal assessment

Upon admission and during the intubation procedure, a thorough examination of the upper airway mucosa was performed. Direct laryngoscopy revealed diffuse mucosal erythema and edema involving the oropharynx and larynx, without ulceration or pseudomembrane formation. Bedside bronchoscopy performed on hospital day 17 due to persistent respiratory concerns further confirmed congested but intact tracheobronchial mucosa, with no evidence of adhesions, stenosis, or granulation tissue. These findings guided subsequent targeted nursing interventions to prevent mucosal complications.

#### 7.4.2 Endotracheal tube cuff management

Prior to each oral care session, endotracheal tube cuff pressure was carefully monitored and maintained between 20 and 30 cmH2O. This ensured an effective seal to prevent aspiration while avoiding ischemic injury to the tracheal mucosa, thereby reducing the risk of ventilator-associated pneumonia (VAP) and long-term tracheal stenosis.

#### 7.4.3 Structured oral cleansing and antiseptic care

Twice daily, coordinated oral care was performed by two nurses. One nurse gently irrigated and cleansed the oral cavity using a sequence of normal saline, sterile water, and diluted povidone-iodine solution (20:1 ratio), while the second nurse performed continuous suctioning to maintain airway safety. Following cleansing, 0.12% chlorhexidine gluconate mouthwash was instilled and retained for 3–5 min before suctioning, to reduce oropharyngeal bacterial colonization.

#### 7.4.4 Perioral and mucosal repair

The denuded perioral skin and intraoral mucosa were treated every 6 h with a 20-minute wet compress using sterile water and povidone-iodine (9:1 ratio). Recombinant bovine basic fibroblast growth factor (bFGF) gel was then evenly applied via petrolatum gauze to promote re-epithelialization.

#### 7.4.5 Mucosal complication prevention across sites

To prevent adhesions and stenosis in the eye, oropharynx, and airway, a consistent approach was adopted: regular lubrication and separation of mucosal surfaces (e.g., using a sterile glass rod for the conjunctival fornices and careful oral swabbing), maintenance of adequate humidification of inspired gases, and minimization of tube movement or traumatic suctioning. These measures were systematically applied alongside systemic anti-inflammatory therapy.

#### 7.4.6 Outcomes

Through this integrated assessment and preventive approach, oral mucosal lesions healed progressively, oral malodor resolved, and no incidence of VAP, mucosal adhesions, or airway stenosis occurred during the ventilatory period or after extubation. This structured mucosal care protocol proved both safe and effective in this high-risk pediatric TEN patient.

### 7.5 Urethral protection

Urogenital involvement in toxic epidermal necrolysis (TEN) carries a significant risk of long-term morbidity. Blistering and erosions of the urethral mucosa, if not managed proactively, may lead to stricture formation and voiding dysfunction (18). In the present case, epidermal denudation was evident in the genital region, indicating probable urethral mucosal involvement. A preventive urethral care protocol was therefore initiated immediately upon admission.

#### 7.5.1 Catheter selection and rationale

A biocompatible silicone Foley catheter was inserted to stent the urethral lumen, thereby preventing contact between denuded mucosal surfaces and subsequent adhesion. This approach also diverted urine away from the denuded perineal skin, reducing chemical irritation and infection risk.

#### 7.5.2 Adhesion prevention

The catheter was gently rotated at its meatal junction every 4 h to prevent adherence to the urethral epithelium.

#### 7.5.3 Perineal hygiene and local wound care

The perineum was cleansed twice daily using a sterile, gentle irrigant. In line with the whole-body skin care protocol, the denuded genital skin was managed with petrolatum gauze and topical recombinant bovine basic fibroblast growth factor (bFGF) gel to promote healing.

#### 7.5.4 Continuous monitoring

Urine output, color, and character were rigorously assessed each nursing shift. Any abnormalities were promptly communicated to the medical team for further evaluation.

#### 7.5.5 Outcome

The silicone catheter remained in place for 22 days and was subsequently removed without complication. No catheter-associated urinary tract infection, urethral stricture, or dysuria occurred during or after hospitalization. This structured preventive strategy effectively averted urological sequelae in this high-risk pediatric patient.

## 8 Discussion

The management of pediatric toxic epidermal necrolysis (TEN) complicated by respiratory failure presents a formidable therapeutic conflict: life-sustaining interventions such as endotracheal intubation inevitably inflict further damage on already fragile skin and mucosa, while inadequate airway support threatens survival (7, 19). This case of a 6-year-old boy with 70% BSA involvement successfully navigated this dilemma through a systematically implemented, multidisciplinary, and phase-based nursing strategy. The uniqueness of our approach lies not merely in combining available interventions, but in structuring them into a coherent protocol that dynamically adapts to the evolving wound biology and the child's changing respiratory needs, thereby transforming a high-risk scenario into a controlled, predictable recovery trajectory.

Our phased skin-care protocol was explicitly aligned with the classic stages of wound healing-inflammatory, proliferative, and remodeling (20, 21). During the inflammatory phase (characterized here by extensive detachment, bleeding, and high exudate), the priority was protection and infection control: petrolatum gauze coverage, saline-povidone-iodine cleansing, and avoidance of adhesive trauma through innovative non-adhesive tube securement (22, 23). As the wounds transitioned into the proliferative phase (evidenced by granulation tissue emergence), the focus shifted to active healing promotion: topical recombinant bovine basic fibroblast growth factor (bFGF) gel was applied directly to the wound bed, supported by infrared therapy to enhance cellular activity and angiogenesis. In the subsequent remodeling phase (marked by eschar formation and re-epithelialization), care evolved toward gentle debridement of crusts, management of hyperpigmentation, and the use of silicone-based dressings to minimize scarring (24, 25). This conscious staging of interventions according to wound physiology ensured that each nursing action was biologically timed to optimize recovery. Notably, this phased approach aligns with the foundational principles of modern burn wound management, which similarly emphasizes stage-appropriate care to optimize epithelialization and minimize scarring (26, 27).

Furthermore, our case underscores the importance of proactive airway mucosal assessment in pediatric TEN with respiratory failure. Bronchoscopic evaluation not only aided in diagnosing ventilator-associated pneumonia but also provided direct visualization of mucosal integrity, guiding targeted interventions to prevent adhesions and stenosis. The integration of regular laryngo-bronchoscopic exams with a structured nursing protocol for mucosal care—including cuff pressure management, humidification, and secretion control—may help mitigate long-term aerodigestive complications in this vulnerable population.

The success of this case was fundamentally underpinned by a structured multidisciplinary team (MDT) model operating under a clear “specialist collaboration with assigned responsibilities” framework. The integration of PICU (life support), dermatology (daily wound assessment and regenerative intervention), infectious diseases (antimicrobial stewardship), and nutrition (tailored metabolic support) ensured that every aspect of the child's critical illness was addressed by experts in parallel. This model holds significant translational potential for the care of other critically ill children with extensive integumentary involvement, such as those with severe burns, major bullous dermatoses, or extensive soft-tissue infections. In pediatric intensive care, where organ support and tissue integrity are often interdependent, a proactive, coordinated MDT approach can standardize care, reduce iatrogenic harm, and improve long-term functional outcomes across a range of complex conditions.

Several limitations of this report must be acknowledged. First, as a single-center case report, the generalizability of our findings is inherently limited; the favorable outcome may be influenced by center-specific expertise and resources. Second, the retrospective nature of the description precludes controlled comparison with alternative management strategies. Future studies should aim to validate this phased, MDT-driven nursing framework through prospective, multi-center cohorts of pediatric TEN or similar severe dermatologic emergencies. Additionally, the development and validation of a risk-assessment tool for skin and mucosal injury progression in critically ill children could help standardize early intervention and further personalize care pathways. Such efforts would strengthen the evidence base for the systematic, biology-informed nursing practice illustrated in this case.

Finally, the follow-up period documented in this report is limited to the inpatient phase. Consequently, we are unable to present the 3-to 6-month post-discharge data specifically requested by reviewers regarding otorhinolaryngological (ENT), ophthalmological, urological, and dermatological sequelae, such as adhesions, strictures, visual acuity changes, and scar maturation. This gap highlights a common challenge in reporting acute critical care cases and underscores the critical importance of structured, multidisciplinary long-term follow-up in TEN. The absence of this data precludes a full assessment of the long-term efficacy of our acute-phase interventions in preventing disabling complications.

In conclusion, this case demonstrates that even in the most severe pediatric TEN complicated by respiratory failure, a carefully structured, phase-aligned, and multidisciplinary nursing strategy can successfully reconcile the imperatives of life support and tissue preservation. The principles outlined—dynamic wound staging, innovative trauma-minimizing techniques, and proactive mucosal protection—offer a replicable framework for managing similar high-risk conditions in the pediatric intensive care setting.

## Data Availability

The original contributions presented in the study are included in the article/Supplementary Material, further inquiries can be directed to the corresponding author.
